# Cyclic Peptide-Capped Gold Nanoparticles for Enhanced siRNA Delivery

**DOI:** 10.3390/molecules190913319

**Published:** 2014-08-28

**Authors:** Amir Nasrolahi Shirazi, Karissa L. Paquin, Niall G. Howlett, Dindyal Mandal, Keykavous Parang

**Affiliations:** 1 School of Pharmacy, Chapman University, Irvine, CA 92618, USA; 2 Department of Cell and Molecular Biology, University of Rhode Island, Kingston, RI 02881, USA

**Keywords:** small interfering RNA (siRNA) delivery, gold nanoparticles, cyclic peptides, siRNA delivery systems (DDS)

## Abstract

Previously, we have reported the synthesis of a homochiral l-cyclic peptide [WR]_5_ and its use for delivery of anti-HIV drugs and biomolecules. A physical mixture of HAuCl_4_ and the peptide generated peptide-capped gold nanoparticles. Here, [WR]_5_ and [WR]_5_-AuNPs were tested for their efficiency to deliver a small interfering RNA molecule (siRNA) in human cervix adenocarcinoma (HeLa) cells. Flow cytometry investigation revealed that the intracellular uptake of a fluorescence-labeled non-targeting siRNA (200 nM) was enhanced in the presence of [WR]_5_ and [WR]_5_-AuNPs by 2- and 3.8-fold when compared with that of siRNA alone after 24 h incubation. Comparative toxicity results showed that [WR]_5_ and [WR]_5_-AuNPs were less toxic in cells compared to other available carrier systems, such as Lipofectamine.

## 1. Introduction

Small interfering RNA (siRNA) is a class of RNA molecules that elicits knockdown of its complimentary target mRNA and consequently inhibits the expression of the corresponding protein [1]. siRNA has been extensively investigated as one of the relatively modern therapeutics in the treatment of various diseases [2,3,4,5,6,7]. However, further clinical investigation of siRNA has been challenging due to their relatively large size, poor intracellular uptake due to the presence of negatively charged phosphate groups in their structure, and enzymatic degradation under *in vivo* environment [8,9]. Thus, several delivery systems, such as polymeric-based [10], cholesterol containing [11], lipid-based like liposomes [12], and carbon nanotubes (CNTs) [13] have been employed to improve several properties of siRNA including limited intracellular delivery, degradation in serum, and off-target effects.

Despite this development, siRNA-mediated delivery systems have not been entirely successful since the majority of delivery systems exhibited significant cytotoxicity and showed limited potential for siRNA delivery in some cell lines [14,15,16,17,18]. For instance, post-mitotic primary neuron cells showed high resistance to lipofectamin [18]. Meanwhile, due to the great therapeutic potential of siRNA, developing efficient delivery systems is highly demanded in clinical investigations. Currently, Lipofectamine has been used as one of the common systems for siRNA delivery. However, in several cases, like E18 rat embryonic cortical and hippocampal cells the transfection efficiency was significantly low (3%–25%) [19].

In last two decades, cell-penetrating peptides (CPPs) have been widely employed as non-viral intracellular carriers by taking advantage of their unparalleled properties like biocompatibility and cell penetration ability to cross the plasma membrane or through endocytic pathways [20]. A wide range of molecular cargos, such as small drugs [21,22,23,24], biologically important molecules [25], and relatively large liposomes [26,27] have been transported by using CPP-mediated delivery methods. Structurally, the majority of CPPs contain positively charged amino acids (e.g., arginine and/or lysine). The presence of positive charge on the surface of CPPs helps them to interact with negatively charged elements (e.g., heparin and/or phosphate groups) in cell membrane structure. This interaction can trigger the internalization of the peptide.

In addition to the cell penetrating capability of CPPs, the molecular cargos loading and release are significantly important in overall functionality of the system. Thus, to date, several CPPs have been discovered that can be employed as transportation tools for the intracellular delivery of different biomolecules through non-covalent interaction. Furthermore, loading molecular cargos through non-covalent interactions offers significant advantages including smooth release of drug in their intact form and simple loading procedure when compared with the covalent methods [28,29]. Technically, amino acids with lipophilic regions like tryptophan can generate a hydrophobic region. The presence of the hydrophobic region can further assist the system to entrap the relatively large biomolecules. The formation of cargo complex-CPP through mostly electrostatic interaction and hydrophobic forces is a simple and easy approach. The complex formation can reform the structure and induce cell permeability to the conformation of relatively large molecules. Furthermore, positively charged portion of CPPs are able to hold the negatively charged backbone in siRNAs structure. These nonspecific electrostatic interactions facilitate the carrier-siRNA complex formation. In addition, other parameters like the molar and/or charge ratio of the CPPs to siRNA can control the functionality of the system. It has been reported that the excess amount of CPPs can facilitate the formation of the CPP-siRNA complex and cover the surface of the complex by positive charge to increase the cell permeability of the system [30]. Moreover, the hydrophobic portion of the CPPs could be responsible for the entrapment of siRNA and disturbs the stabilization of the plasma membrane. Recently, several *in vivo* investigations have reported by employing the non-covalent approach between CPP and siRNA [31].

During the last few years we have been investigating the application of cyclic peptides in the delivery of a wide range of cargos (including anticancer agents, anti-HIV drugs, and negatively charged phosphopeptides). The cyclic conformation of the peptide offers higher cell penetrating ability and stability over linear counterparts. Our studies showed that the intracellular uptake of several molecular cargos can be significantly enhanced in the presence of a cyclic peptide containing alternative arginine and tryptophan namely [WR]_5_ [20,32,33]. Moreover, the formation of [WR]_5_-capped gold nanoparticles were discovered to improve the intracellular delivery of model anti-*HIV* drugs and phosphopeptides [34,35]. In continuation of our efforts to develop new applications of cyclic peptide-based drug delivery systems for transporting biomolecules, we report here the use of [WR]_5_- and [WR]_5_-AuNPs for improving the cellular delivery of siRNA. To the best of our knowledge, this is the first report of using cyclic peptide-based delivery systems for cellular transporting of siRNA.

## 2. Results and Discussion

### 2.1. Synthesis of the Cyclic Peptide

**Scheme 1:**
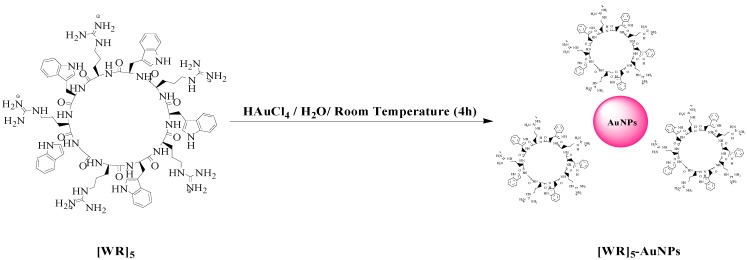
Chemical structures of [WR]_5_ and [WR]_5_-AuNPs.

**Scheme 2:**
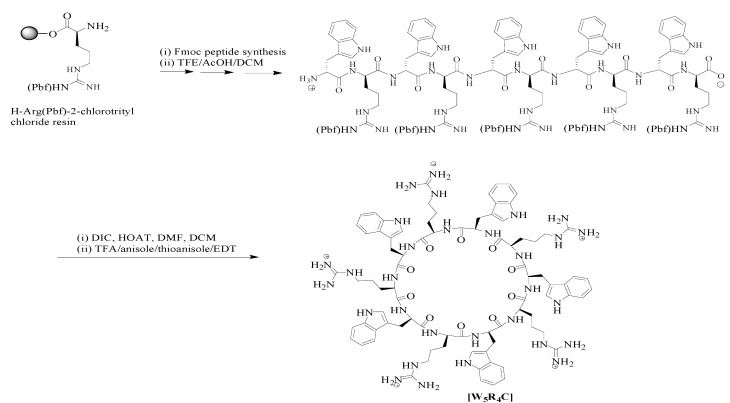
Solid-phase synthesis of cyclic [WR]_5_.

A cyclic decapeptide containing alternative l-arginine and l-tryptophan (Scheme 1) was synthesized by using 9-fluorenylmethyloxycarbonyl (Fmoc)-based chemistry based on our previously reported method [20,32,33]. The peptide was designed based on the presence of hydrophobic tryptophan and positively charged arginine. The presence of positively charged arginine residues is critical since they can trigger electrostatic interactions with negatively charged elements in the cell membrane, such as phosphate groups and heparin residues. Furthermore, tryptophan helps to generate a hydrophobic region and facilitate the transportation of siRNA through disturbing the phospholipid bilayer and leads to a higher uptake. The combination of tryptophan and arginine residues induced the reaction of the peptide with HAuCl_4_ solution. Mechanistically, tryptophan has been used for the formation of gold nanoparticles since this amino acid can reduce Au^3+^ into Au°. Moreover, arginine attracts the negatively charged charged aurate anions into the reaction site [24]. Thus, this mixure of amino acids could be an optimized arrangement to increase the yield of gold nanoparticles synthesis, and an efficient collection for cellular uptake enhancement (Scheme 2).

### 2.2. Synthesis of Peptide-Capped Gold Nanoparticles

The synthesis of [WR]_5_-AuNPs was performed by an *in situ* method. The peptide was dissolved in water (1 mM) and mixed with an aqueous solution of HAuCl_4_ (1 mM). The peptide-capped nanoparticles were obtained after 4 h incubation at room temperature. The final concentrations of gold and peptides were adjusted to be 500 μM. The purple color showed the formation of CPP-AuNPs. Mechanistically, the CPP works as reducing and capping agents to generate peptide capped AuNPs in one step. The synthesized peptide-capped gold nanoparticles were characterized by different techniques such as UV-Vis spectroscopy, transmission electron microscopy (TEM), and dynamic light scattering (DLS) techniques as previously reported by us [35]. The formation of synthesized nanoparticles was confirmed as their characteristic peak showed up at 540 nm (Figure S1). Scanning electron microscopy (SEM) images showed that nanoparticles formed in a size range of 200–230 nm (Figure S2). DLS also validated the size of nanoparticles. Furthermore, zeta potential was used to measure the charge on the surface of nanoparticles. The elevation in zeta potential of [WR]_5_ and [WR]_5_-AuNPs were determined to be +56.2 ± 7.6 mv and +18.35 ± 2.5 mv respectively. These data showed that the positively charged arginine residues can cover the surface of nanoparticles through electrostatic interactions.

### 2.3. Cytotoxicity of siRNA Delivery Systems

**Figure 1:**
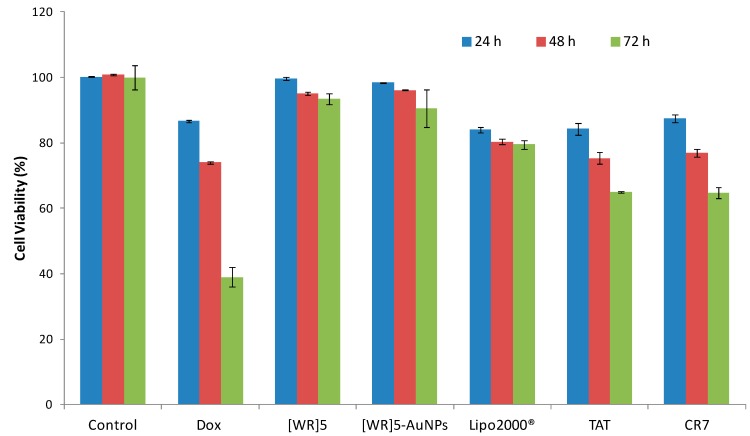
Cytotoxicity of the [WR]_5_ and corresponding peptide-capped AuNPs compared to other siRNA delivery systems.

Initially, the peptide and the corresponding peptide capped-gold nanoparticles were tested for their toxicity in human cervical adenocarcinoma (HeLa) cells. We compared the cytotoxicity of [WR]_5_ and [WR]_5_-AuNPs CPPs with transfection reagents, such as polyarginine (CR_7_), TAT (YGRKKRRQRRRC), and Lipofectamine (Lipo2000^®^, a cationic lipid formulation) at different incubation times (24–72 h). As it is shown in Figure 1, [WR]_5_-AuNPs (25 μM) and [WR]_5_ (25 μM) exhibited significantly lower toxicity in HeLa cells compared to other CPPs like polyarginine (25 μM), TAT (25 μM), and Lipofectamine (200 nM) by 21%–55%. Based on the cytotoxicity results, the noncytotoxic concentration of 25 μM was chosen for further studies.

### 2.4. Cellular Uptake of F′-siRNA by Flow Cytometry

**Figure 2:**
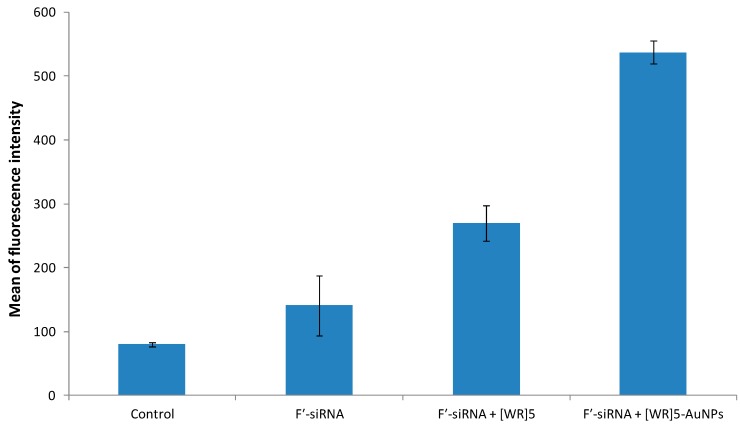
Cellular uptake of F′-siRNA in the presence and absence of [WR]_5_ and [WR]_5_-AuNPs after 4 h incubation.

To evaluate [WR]_5_-AuNPs and [WR]_5_ as siRNA transporters, a model experiment with non-targeting fluorescent siRNA, F′-siRNA (where F′ = 5,6-carboxyfluorescein (FAM), GenePharma, Shanghai, China) as a cargo molecule was performed. This siRNA with a sequence of 5'-UUC UCC GAA CGU GUC ACG UTT-3' was selected as a model cargo to determine the efficiency of the system. Due to the presence of the negatively charged segments in the structure of siRNA, an efficient cellular uptake is critical for its activity. HeLa cells were incubated with F′-siRNA (200 nM) in the presence or absence of transporters [WR]_5_-AuNPs and [WR]_5_ (25 μM) for 24 h at 37 °C and then treated with trypsin to remove cell surface-bound siRNA. Intracellular uptake of F′-siRNA was measured in cells using fluorescence activated cell sorter (FACS). FACS showed higher fluorescence signal in cells treated with F′-siRNA-loaded [WR]_5_ and [WR]_5_-AuNPs compared to that of F′-siRNA alone. For instance, the cellular uptake of F′-siRNA-loaded [WR]_5_-AuNPs and F′-siRNA-loaded [WR]_5_ were found to be approximately 3.8- and 2-fold higher for F′-siRNA than those of F′-siRNA alone, respectively (Figure 2), suggesting that the uptake of siRNA is facilitated by the carriers. These data also suggest that a [WR]_5_ and their AuNPs can enhance cellular association of the siRNA. Peptide-capped gold nanoparticles showed higher efficiency in delivering siRNA into cells compared to the peptide alone. Both AuNPs and [WR]_5_ have been reported as cellular delivery agents. The combination of both in peptide-capped AuNPs will generate nanoparticles with more transfection efficiency. AuNPs have highly active surface for binding to siRNA. The formation of peptide-capped AuNPs changes the orientation of amino acids in the structure of the peptide and enhances their efficiency. 

### 2.5. Cellular Uptake of F′-siRNA by Microscopy

**Figure 3:**
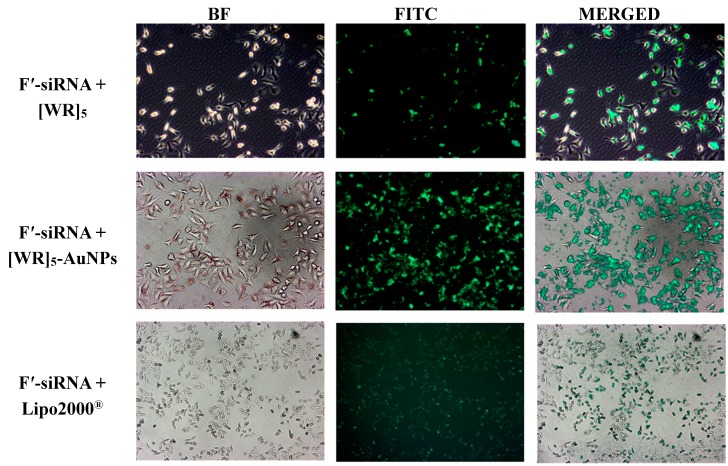
Fluorescence microscope images of F′-siRNA uptake by HeLa cells in the presence of [WR]_5_ and [WR]_5_-AuNPs after 4 h incubation. No green fluorescence was observed in the presence of F′-siRNA alone. BF: Brightfield, FITC: Fluorescein isothiocyanate.

To confirm the improvement of cellular uptake of the siRNA, the fluorescence intensity of F′-siRNA was visualized in HeLa cells by microscopy techniques. Microscopy results exhibited the localization of F′-siRNA-loaded [WR]_5_-AuNPs and F′-siRNA-loaded [WR]_5_. Lipofectamine was used as a control for microscopy studies. We found that the presence of Lipo2000^®^ enhanced the fluorescence signal in cells suggesting the intracellular uptake of the F′-siRNA. However, higher intensity was observed in the presence of [WR]_5_-AuNPs compared to that of Lipo2000^®^, suggesting that this lipid-based reagent did not work as efficiently as the peptide-capped AuNPs. These data confirmed that the presence of the peptide and peptide-capped AuNPs are critical for the enhanced cellular permeability of the molecular cargo (Figure 3).

### 2.6. Cellular Uptake of F'-[WR]_5_-AuNPs

Previously, the procedure for the synthesis of F'-[WR]_5_-AuNPs was reported by us [35]. The mechanistic investigations showed that the uptake of F'-[WR]_5_-AuNPs by cells did not change when endocytic inhibitors were used (Supporting Information, Figure S3). However, EIA had a minor effect on the uptake suggesting that lipid-raft-dependent micropinocytosis and phagocytosis pathways could be slightly responsible for transporting the drug across the cell membrane.

## 3. Experimental Section

### 3.1. General Methods

All reagents were purchased from Wilkem Scientific (Pawtucket, RI, USA). Other chemicals and reagents were purchased from Sigma-Aldrich Chemical Co. (Milwaukee, WI, USA). Coupling reagents, trityl chloride resin, and Fmoc-amino acid building blocks were purchased from Chem-Impex International Inc. (Wood Dale, IL, USA). The non-targeting fluorescent labeled siRNA (FAM-siRNA) was purchased from GenePharma. The sequence osf the siRNA was 5'-UUC UCC GAA CGU GUC ACG UTT-3'. The siRNA against FANCD2 was purchased from Ambion (Grand Island, NY, USA) with a sense sequence of 5'-CAG CCA ACC UGA GAU CCU ATT-3', and antisense of 5′-UAG GAU CUC AGG UAG GCU GGT-3'.

### 3.2. Synthesis of [WR]_5_

The cyclic peptide synthesis was performed by our previously reported procedure [32,33]. Bio-Rad polypropylene columns were used as reaction vessels, and all reactions were carried out by shaking and mixing in a Glass-Col small tube rotator under N_2_ gas at room temperature. For peptide synthesis, solid-phase method employing *N*-(9-fluorenyl)methoxycarbonyl (Fmoc)-based chemistry and Fmoc-l-amino acid building blocks were used. 2-(1*H-*Benzotriazole-1-yl)-1,1,3,3-tetramethyluronium hexafluoro phosphate (HBTU) and *N*,*N*-diisopropylethylamine (DIPEA) were used for coupling and activating amino acids respectively in *N*,*N*-dimethylformamide (DMF). Fmoc deprotection was carried out by using piperidine in DMF (20% v/v). A mixture of trifluoroethanol (TFE)/acetic acid/dichloromethane (DCM) (2:2:6, v/v/v, 15 mL) was used for cleaving the side chain protected peptide from the resin for 2 h. The resin was filtered off, and the solvent was evaporated to collect the linear peptide with side chain protecting groups. To synthesize the cyclic peptide from the linear one, a mixture of 1-hydroxy-7-azabenzotriazole (HOAt, 163.3 mg, 1.2 mmol) and *N*,*N′*-diisopropylcarbodiimide (DIC, 187.0 µL, 1.2 mmol) in dry DMF (100 mL) and dry DCM (40 mL) for 18 h was used. The deprotection of peptide side chains was carried by using a mixture of trifluoroacetic acid (TFA)/thioanisole/anisole/1,2-ethanedithiol (EDT) (90:5:2:3 v/v/v/v) for 2 h. Cold diethyl ether (Et_2_O) was used to precipitate the peptide. The crude peptide was purified by reversed-phase Hitachi HPLC (L-2455) on a Phenomenex Prodigy 10 μm ODS reversed-phase column (2.1 cm × 25 cm). The peptide was purified by eluting the crude peptide at 10.0 mL/min using a gradient of 0%–100% acetonitrile (0.1% TFA) and water (0.1% TFA) over 60 min. The peptide was then lyophilized. A high-resolution Biosystems QStar Elite time-of-flight electrospray mass spectrometer and a MALDI AXIMA Performance TOF/TOF mass spectrometer (Shimadzu, Tokyo, Japan) were employed to confirm the chemical structure of [WR]_5_ as described previously [21,22]. Analytical HPLC was used to confirm the purity of final products (>95%, Figure S4). The analytical HPLC was performed on Thermo Hypersil Gold C18 analytical column (150 × 4.6 mm). 

### 3.3. Spectral Data

*[WR]_5_*: HR-MS (ESI-TOF) (*m/z*): C_85_H_110_N_30_O_10_: calcd, 1710.9021; found, 1711.6974 [M+H]^+^, 571.5658 [M+3H]^3+^, 428.9286 [M+4H]^4+^, 343.1493 [M+5H]^5+^.

### 3.4. Synthesis of Peptide-Capped Gold Nanoparticles

The synthesis of AuNPs was carried out by using [WR]_5_ peptide and visual evaluation technique. Mixing HAuCl_4_ (1 mM) and [WR]_5_ (1 mM) solutions led to the formation of peptide-capped AuNPs. The cyclic peptide was dissolved in deionized water (1 mL) to prepare the stock solution (1 mM). The stock solution (1 mL, 1 mM) was mixed with HAuCl_4_ solution (1 mL, 1 mM) to synthesize AuNPs. The color of the solution was turned into purple upon the formation of AuNPs. Subsequently, all concentrations in cell based assays was calculated based on the concentration of the peptide solution and HAuCl_4_ solution.

### 3.5. Scanning Electron Microscopy (SEM)

To prepare a sample for SEM microscopy, [WR]_5_-AuNPs (5 μL of 0.5 mM solution in H_2_O) solution was spotted onto a carbon-coated copper grid (300 mesh). The liquid drop was then allowed to stay on the carbon film for 10 min. The excess of the solution was removed from the surface of the grid, and the sample was kept overnight to get dried. SEM analyses of [WR]_5_-AuNPs were conducted in an FEI Nova NanoSEM (Hillsboro, OR, USA) using the directional backscatter (DBS) electron detector. 

### 3.6. Cell Culture

Human cervix adenocarcinoma (HeLa) cells (ATCC no CCL-2) was obtained from the American Type Culture Collection (Manassas, VA, USA). Cells were grown on 75 cm^2^ cell culture flasks with EMEM medium, supplemented with 10% fetal bovine serum (FBS), and 1% penicillin-streptomycin solution (10,000 units of penicillin and 10 mg of streptomycin in 0.9% NaCl) in a humidified atmosphere of 5% CO_2_, 95% air at 37 °C. 

#### 3.6.1. Cytotoxicity Assay

The cytotoxicity assay was performed through MTS cell viability assay. HeLa cells (4000) were seeded in 0.1 mL per well in 96-well plates 24 h prior to the experiment. The old medium (EMEM containing FBS (10%)) was changed by compounds including [WR]_5_ (25 μM), [WR]_5_-AuNPs (25 μM), Lipofectamine (2 µg/mL), polyarginine (25 μM), and TAT peptide (25 μM) in serum containing medium and incubated for 24–72 h at 37 °C in a humidified atmosphere of 5% CO_2_. 20 μL of CellTiter 96 aqueous solution (Promega, Madison, WI, USA) was added. Cell viability was calculated with the fluorescence intensity at 490 nm using a SpectraMax M2 microplate spectrophotometer. The percentage of cell survival was calculated as [(OD value of cells treated with the test mixture of compounds) − (OD value of culture medium)]/[(OD value of control cells) − (OD value of culture medium)] × 100%.

#### 3.6.2. Microscopy Imaging

HeLa cells were grown with antibiotic-free DMEM 24 h prior to the experiment in 6-well plates (2 × 10^5^ cells in 1 mL media per well). The FAM-siRNA was incubated with [WR]_5_, [WR]_5_-AuNPs, or Lipofectamine for 30 min at room temperature. Then, the cells were treated with a mixture of FAM-siRNA (200 nM) and [WR]_5_ (25 μM), [WR]_5_-AuNPs (25 μM), or Lipofectamine (2 μg/mL) in Opti-MEM for 4 h and 24 h at 37 °C. After a specific incubation time, the media containing the treatments was removed, and PBS was added to cells. Microscopy was carried out using a Zeiss Axio Imager (Oberkochen, Germany) A1 and Vision Rel. 4.6, Dell Optiplex 735. The imaging was carried out under fluorescence and bright field at 50×, 100×, and 400× magnifications. Images were merged using Adobe Photoshop in order to visualize the localization of fluorescence. 

#### 3.6.3. Flow Cytometry

HeLa cells were taken in 6-well plates with the population of 3 × 10^5^ cells per well in opti-MEM. Then, the fluorescence-labelled siRNA (FAM-siRNA, F′-siRNA, 200 nM) alone and in combination with [WR]_5_ (25 μM) and [WR]5-AuNPs (25 μM) were added to the different wells in serum with no media. The plates were incubated for 4 h at 37 °C. As a negative control, wells with no treatment and F′-siRNA alone were used. After 4 h incubation, the media containing the treatments were removed. The cells were digested with 0.25% trypsin/ EDTA (0.53 mM) for 5 min to remove any artificial surface binding. Then the cells were washed twice with PBS. Finally, the cells were resuspended in flow cytometry buffer and analyzed by flow cytometry (FACSVerse flow cytometer, San Jose, CA, USA) using FITC channel and CellQuest software. The data presented were based on the mean fluorescence signal for 10,000 cells collected. All assays were performed in duplicate.

#### 3.6.4. Mechanism of Cellular Uptake when Endocytic Inhibitors Are Used

Cells were kept in 6-well plates prior to performing the assay. All wells containing cells were incubated with endocytic inhibitors 30 min before the treatment. Several inhibitors like methyl-β-cyclodextrin (2.5 mM), chloroquine (100 μM), nystatin (50 μg/mL), 5-(*N*-ethyl-*N*-isopropyl)amiloride (EIA, 50 μM), and chlorpromazine (30 μM) were used. The media was removed and cells were incubated with a combination of F′-[WR]_5_-AuNPs and inhibitors for 4 h. Similar FACS analysis method was used to evaluate the data.

## 4. Conclusions

In conclusion, a new class of cyclic peptide and corresponding cyclic peptide-capped AuNPs containing arginine and tryptophan were evaluated for the delivery of siRNA. They showed to be efficient in the intracellular delivery of F′-siRNA. Furthermore, [WR]_5_-AuNPs showed negligible toxicity up to 100 μM in HeLa cells. However, the concentration of 25 µM was used for cell-based assays. [WR]_5_-AuNPs was able to function as a transporter of fluorescence-labeled siRNA and intracellularly. The high cellular internalization of the labeled drugs by [WR]_5_-AuNPs suggests the potential application of these nanoparticles as a molecular transporter. The present results provide preliminary insights for further optimization in degradation, binding, and release properties of cyclic peptide-based carriers in this class as siRNA delivery transporters. 

## Supplementary Materials

Supplementary materials can be accessed at: http://www.mdpi.com/1420-3049/19/9/13319/s1.

## Supplementary Files

Supplementary File 1
