# Supplementary material for: Cyclic Peptide-Capped Gold Nanoparticles for Enhanced siRNA Delivery
Source: Molecules. 2014 Aug 28;19(9):13319–31. doi: 10.3390/molecules190913319 (PMC6271229; doi:10.3390/molecules190913319)
Supplement: Supplementary File 1 [file molecules-19-13319-s001.pdf]

Supplementary Materials

Analytical HPLC

The analytical HPLC was performed on a Thermo Hypersil Gold C18 analytical column (150 × 4.6 mm) using a gradient solvent system, and a flow rate of 0.4 mL/min with detection at 220 nm.

Table S1. HPLC Gradient System.

Time (min)	Water-A (%)	Acetonitrile-B (%)	Flow Rate (mL/min)
0.0	100.0	0.0	1.0
10.0	0.0	100.0	1.0
14.0	100.0	0.0	1.0
16.0	100.0	0.0	1.0

Figure S1. UV-Vis peak of $[WR]_5$-AuNPs. Reprinted with permission from from Shirazi, N.A., *et al.*, Cyclic peptide-capped gold nanoparticles as drug delivery systems; published in *Molecular Pharmaceutics*, Copyright 2013 American Chemical Society [1].

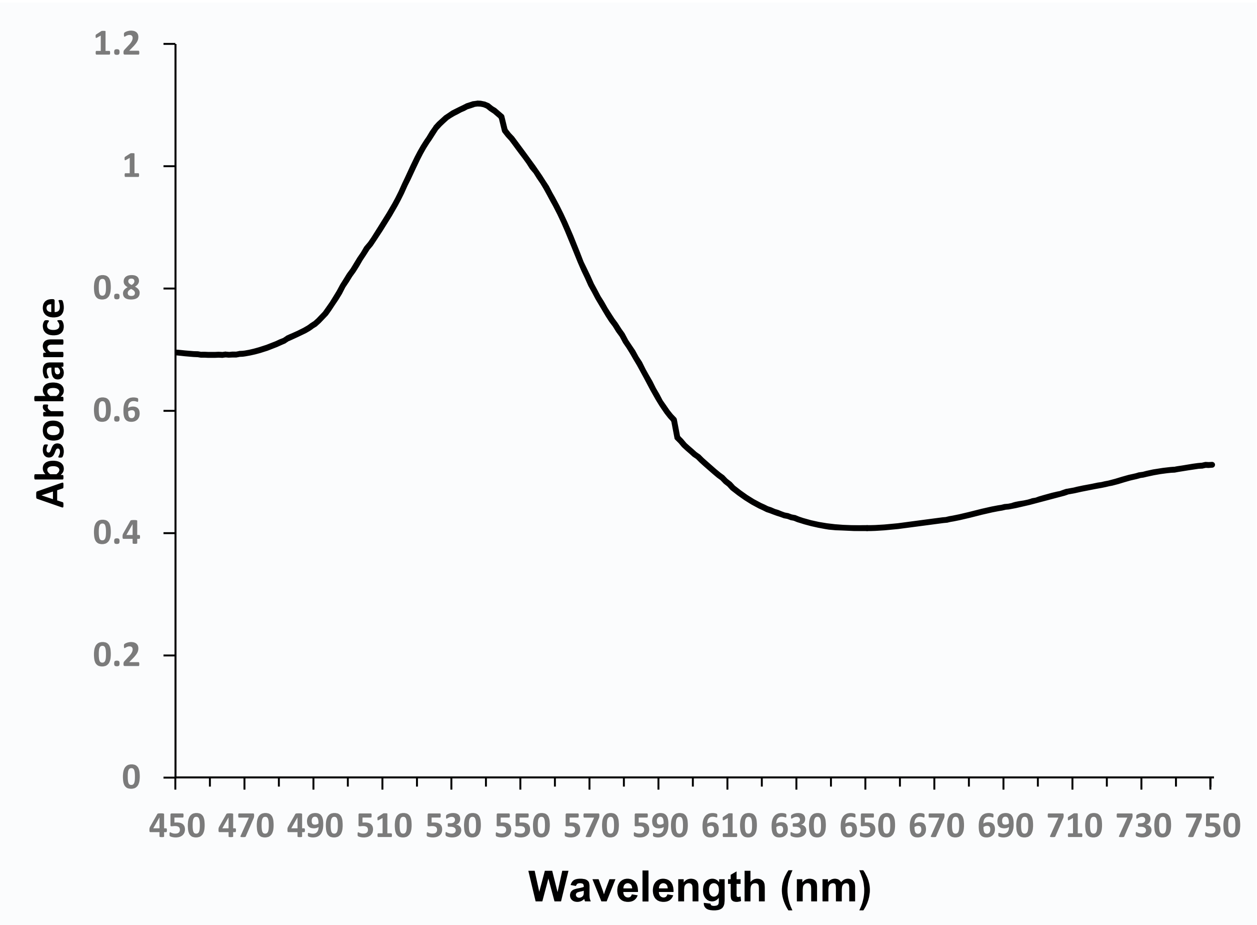

Figure S2. SEM image of [WR]5-AuNPs. Reprinted with permission from Shirazi, N.A., *et al.*, Cyclic peptide-capped gold nanoparticles as drug delivery systems; published in *Molecular Pharmaceutics*, Copyright 2013 American Chemical Society [1].

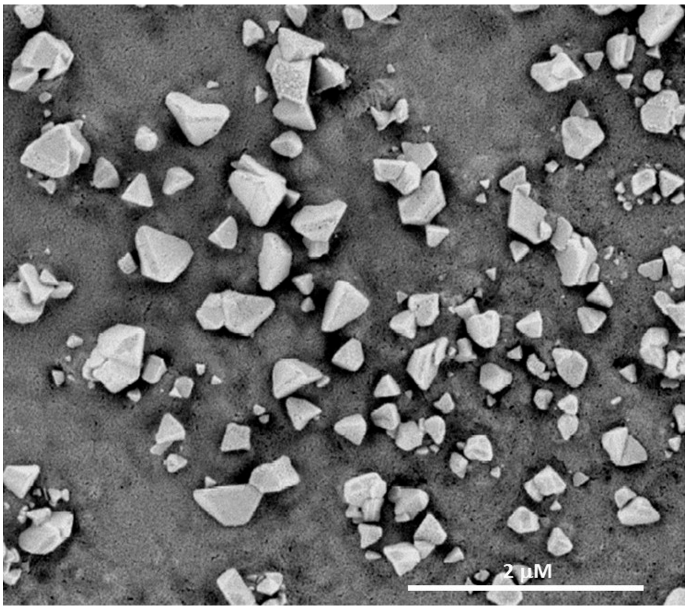

Figure S3. Uptake of F′-[WR]5-AuNPs by cells after 4 h when endocytic inhibitors used.

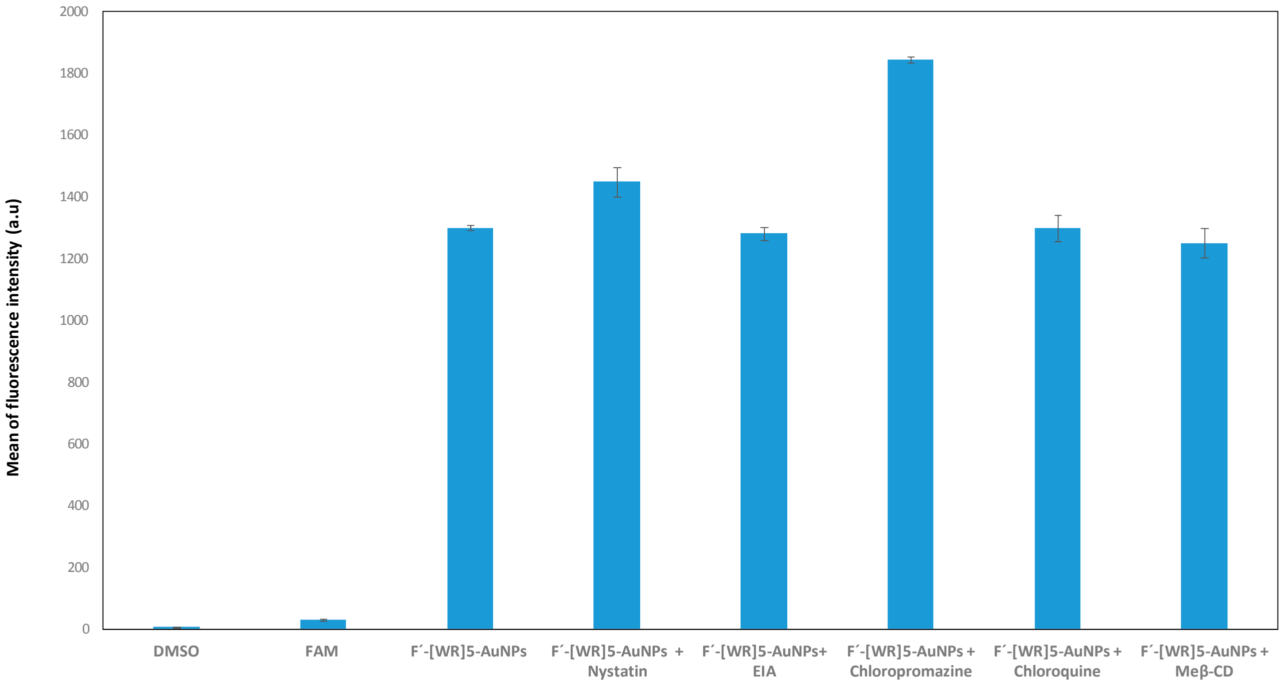

Figure S4. Analytical HPLC chromatogram of $[WR]_5$.

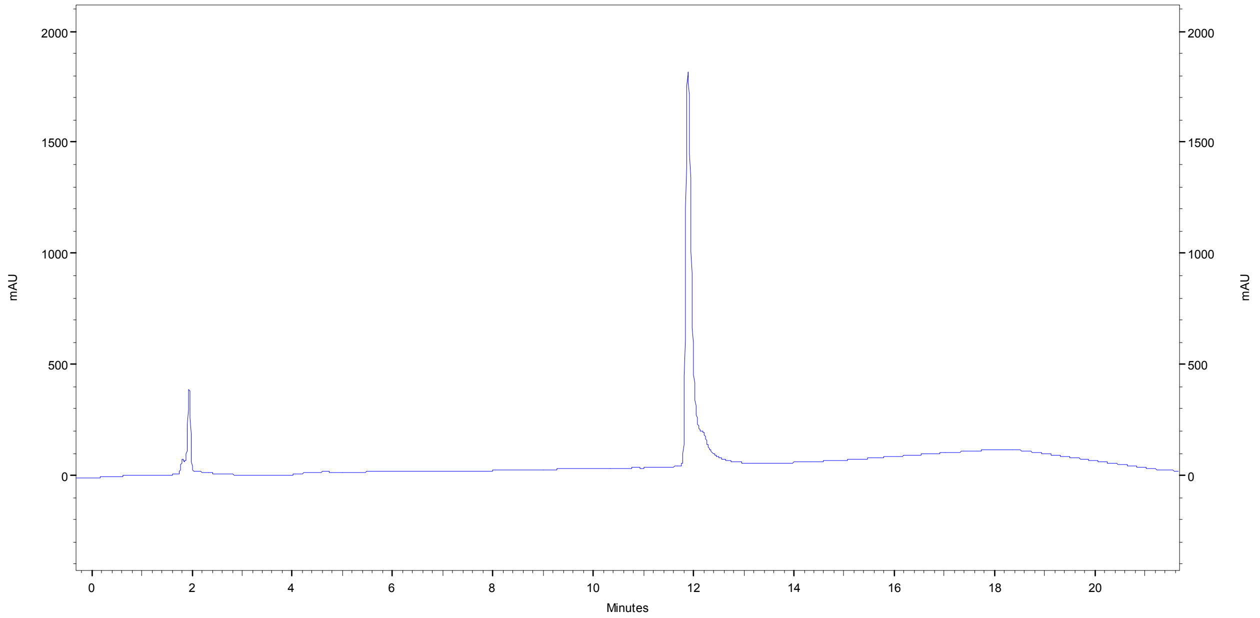

min

Reference

1. Shirazi, N.A.; Mandal, D.; Tiwari, R.K.; Guo, L.; Lu, W.; Parang, K. Cyclic peptide-capped gold nanoparticles as drug delivery systems. *Mol. Pharm.* **2013**, *10*, 500–511.
